# Disrupted balance between pro-inflammatory lipid mediators and anti-inflammatory specialized pro-resolving mediators is linked to hyperinflammation in patients with alcoholic hepatitis

**DOI:** 10.3389/fimmu.2024.1377236

**Published:** 2024-11-21

**Authors:** Wei Li, Ying Xia, Jing Yang, Arun J. Sanyal, Vijay H. Shah, Naga P. Chalasani, Qigui Yu

**Affiliations:** ^1^ Department of Microbiology and Immunology, Indiana University School of Medicine, Indianapolis, IN, United States; ^2^ Division of Gastroenterology and Hepatology, Department of Medicine, Virginia Commonwealth University, Richmond, VA, United States; ^3^ Division of Gastroenterology and Hepatology, Mayo Clinic, Rochester, MN, United States; ^4^ Division of Gastroenterology and Hepatology, Department of Medicine, Indiana University School of Medicine, Indianapolis, IN, United States

**Keywords:** alcoholic hepatitis, specialized pro-resolving mediator, lipid mediator, longitudinal study, alcohol abstinence

## Abstract

**Background:**

Alcoholic hepatitis (AH) is characterized by intense systemic and liver inflammation, posing significant risks of health complications and mortality. While inflammation is a crucial defense mechanism against injury and infection, its timely resolution is essential to prevent tissue damage and restore tissue homeostasis. The resolution of inflammation is primarily governed by specialized pro-resolving mediators (SPMs), lipid metabolites derived from w-6 and w-3 poly-unsaturated fatty acids (PUFAs). Currently, the balance between pro-inflammatory lipid mediators (PLMs) and SPMs in the w-6 and w-3 PUFA metabolic pathways and the impact of alcohol abstinence on profiles of PLMs and SPMs in AH patients are not well studied.

**Methods:**

In this study, we used LC-MS/MS and ELISA to quantify levels of lipid mediators (LMs) and their precursors in the plasma samples from 58 AH patients, 29 heavy drinkers without overt liver diseases (HDCs), and 35 healthy controls (HCs). Subsequently, we assessed correlations of altered LMs with clinical parameters and inflammatory mediators. Furthermore, we conducted a longitudinal study to analyze the effects of alcohol abstinence on LMs over 6- and 12-month follow-ups.

**Results:**

AH patients exhibited significantly higher plasma levels of w-6 PLMs (PGD2 and LTB4) and SPM RvE1 compared to HDCs or HCs. Conversely, the SPM LXA4 was significantly downregulated in AH patients. Some of these altered LMs were found to correlate with AH disease severity and various inflammatory cytokines. Particularly, the LTB4/LXA4 ratio was substantially elevated in AH patients relative to HDCs and HCs. This altered ratio displayed a positive correlation with the MELD score. Importantly, the majority of dysregulated LMs, particularly PLMs, were normalized following alcohol abstinence.

## Introduction

Long-term heavy alcohol consumption leads to a spectrum of alcohol-associated liver diseases (ALD), including alcoholic hepatitis (AH), steatosis, and liver fibrosis/cirrhosis. AH, a critical and progressive acute-on-chronic liver disorder, is characterized by heightened hepatic and systemic inflammation, and is associated with significant morbidity and mortality. Circulating levels of various inflammatory mediators, such as proinflammatory cytokines (TNF-α, IL-6, and IL-8), are profoundly elevated in AH patients and directly correlate with disease severity and mortality rates (1–4). These inflammatory mediators primarily originate from persistently hyperactivated immune cells present in both the peripheral blood and the liver of AH patients (4–6). Alcohol disrupts gap junction integrity of gut mucosal epithelial cells, leading to increased permeability of the gastrointestinal (GI) tract and translocation of microbial components such as lipopolysaccharides (LPS) from the GI tract into the bloodstream and liver (7–11). Alcohol-induced microbial translocation (MT) has been recognized as a major driver of chronic hepatic and systemic inflammation and immune activation in AH patients (8, 9, 12, 13). Additionally, alcoholic metabolites such as acetate, reactive oxygen species (ROS), and acetaldehyde can directly provoke inflammation (14). These inflammatory responses, combined with hyperimmune activation, play a central role in the development of liver fibrosis/cirrhosis, multiple organ failure, and death in AH patients.

While inflammation is a vital defense mechanism against injury and infection, its timely resolution is crucial to prevent tissue damage and restore tissue homeostasis. The inflammatory response comprises two distinct phases: initiation and resolution. In the initiation phase, leukocytes are recruited to injured tissue in response to inflammatory cues, further amplifying the inflammatory response by producing a plethora of proinflammatory mediators. Resolution of inflammation is an actively regulated process, largely governed by specialized pro-resolving mediators (SPMs), including lipoxins (LXs), resolvins (Rvs), protectins, and maresins (MaRs). These SPMs are synthesized from long-chain omega-3 (ω-3) and ω-6 polyunsaturated fatty acids (PUFAs) that are sourced from dietary elements such as meat, eggs, and fish oils (15–18), or derived from ingested short-chain ω-3 (α-linolenic acid, ALA) and ω-6 (linolenic acid, LA) PUFAs from vegetables, plant oils, and seeds (19). Notably, the long-chain ω-6 PUFA, arachidonic acid (AA), serves as a precursor to several potent pro-inflammatory lipid mediators (PLMs), including prostaglandins (PGs), leukotrienes (LTs), and thromboxanes (Txs). AA is converted by cyclooxygenase (COX)-1 and -COX-2 to PGs (PGD2 and PGE2) and Txs and by 5-lipoxygenase (5-LOX) to LTs (e.g. LTB4), leading to initiation of the inflammatory response (15–18). AA can also be converted by 5-, 12-, and 15-LOX to anti-inflammatory LXs (LXA4 and LXB4). Long-chain ω-3 PUFAs such as eicosatetraenoic acid (EPA) and docosahexaenoic acid (DHA) primarily give rise to anti-inflammatory SPMs. EPA ω-3 PUFAs is converted to the E-series Rvs (RvE1 and RvE2) by 15-LOX and 5-LOX (15–18), while DHA ω-3 PUFA is converted by 15-LOX and 5-LOX to D-series Rvs (RvD1-6) and protectins (PD1, PDX), and by 12-LOX/15-LOX type 1 to MaRs (MaR1 and MaR2) (15–18). Importantly, the gene expression, activity, and intracellular location of these enzymes can be regulated by a variety of factors such as inflammatory mediators (20–23), leading to different biosynthetic outcomes of SPMs versus PLMs (17).

SPMs have been detected in both the circulation and tissues of humans in health and disease (24–32). SPMs actively trigger cardinal signals for inflammation resolution through (1) counter-regulation of proinflammatory mediators, (2) promotion of polymorphonuclear cell/neutrophil (PMN) clearance, (3) promotion of phagocytosis to eliminate apoptotic cells and cellular debris, (4) production enhancement of anti-inflammatory cytokines such as IL-10, (5) mitigation of oxidative stress (OS), and (6) modulation of immune cell activation (15, 16, 18, 20, 33–45). However, it remains largely unexplored whether the biosynthetic balance between PLMs and SPMs is disrupted in AH patients, potentially contributing to hyperinflammation, and whether alcohol abstinence restores this balance, thereby reversing hyperinflammation, as AH is usually reversible if individuals successfully abstain from alcohol or undergo medical interventions (46). In this study, we assessed the profiles of circulating PLMs and SPMs in AH patients, comparing them with matched heavy drinkers without overt liver diseases (HDCs) and healthy controls (HCs) (4). Additionally, we monitored changes over 6- and 12-month follow-up intervals of alcohol abstinence. Furthermore, we correlated the altered lipid mediators (LMs) with patients’ clinical parameters, disease severity, levels of inflammatory mediators, as well as markers of MT such as LPS, soluble CD14 (sCD14), and soluble CD163 (sCD163), along with the systemic inflammation marker (C-reactive protein, CRP). To gain deeper insights, we performed data mining to analyze the expression of genes encoding critical enzymes involved in the biosynthesis of LMs in the peripheral blood and liver tissue of AH patients and HCs.

## Materials and methods

### Study subjects and blood samples

The study included 58 AH patients and 29 HDCs at baseline, along with 13 and 9 abstinent AH patients at 6- and 12-month follow-ups, respectively. These subjects were part of a well-characterized cohort enrolled in the multicenter, prospective observatory Translational Research and Evolving Alcoholic Hepatitis Treatment 001 study (TREAT 001, NCT02172898) (47). Demographic and clinical characteristics, as well as drinking patterns of the study subjects, are provided in **Table 1** and **Supplementary Table 1**. Definitions of AH and HDC, along with the inclusion and exclusion criteria, have been previously described (47). In brief, AH was defined by hyperbilirubinemia (amended to >3 mg/dL from >2 mg/dL) and elevated AST levels (>50 IU/L), in the absence of an alternate cause, in individuals with a history of heavy alcohol consumption for at least 6 months, and with the last drink consumed within 6 weeks prior to presentation. In cases where the diagnosis was uncertain, a liver biopsy was performed for confirmation. HDCs were age- and gender-matched individuals with a similar history of alcohol use but without overt clinical liver disease. They were characterized by AST <50 U/L, ALT <50 U/L, and normal total bilirubin levels. All participants were advised to abstain from alcohol and were followed up at 6 and 12 months or until death. The minimum age for participation was 21 years.

**Table 1:** Characteristics of the study cohort.

Variables	Participants			*p* value
	HC (n=35)	AH (n=58)	HDC (n=29)	
Demographics				
Age (years)	42 (26-48)	45 (34-53)	47 (34-53)	0.12
Gender (number and % Male)	18 (51%)	29 (50%)	19 (66%)	0.37
Clinical parameters				
Creatinine (mg/dL)	0.86 (0.69-1.06)	0.81 (0.62-1.10)	0.82 (0.68-1.04)	0.86
Total bilirubin (mg/dL)	0.5 (0.3-0.6)^###^	15.0 (7.8-23.7)***	0.5 (0.4-0.7)	**<0.001**
ALT (IU/L)	9 (7-16)^###^	43 (29-60)***	22 (16-31)^$$^	**<0.001**
AST (IU/L)	17 (14-20)^###^	110 (80-149)***	25 (17-34)	**<0.001**
Prothrombin time (INR)		1.81 (1.60-2.23)***	0.97 (0.92-1.03)	**<0.001**
MELD score		25 (21-28)***	7 (6-8)	**<0.001**
Neutrophils (X10^3^/ml)		8.4 (5.5-15.6)***	3.7 (2.6-4.6)	**<0.001**
Platelets (X10^3^/ml)		153 (105-227)***	244 (197-281)	**<0.001**
BMI		27.2 (24.3-32.2)*	31.5 (26.4-35.7)	**0.05**
Treated with PDN (number and %)		34 (60%)^a^		
30-day mortality (number and %)		4 (6.9%)		
6-month mortality		8 (13.8%)		
12-month mortality		9 (15.5%)		
Drinking patterns				
Total drinking days in 30 days		27 (13-30)	27 (22-30)	0.72
Drinks/day		6 (2-11)	9 (6-14)	0.06

Data are represented as median and interquartile ranges or number and %. HC, healthy controls; AH, patients with alcoholic hepatitis; HDC, heavy drinking controls; ALT, alanine aminotransferase; AST, aspartate aminotransferase; INR, international normalized ratio; MELD, model for end-stage liver disease; PDN, prednisone; BMI, body mass index. Kruskal-Wallis test with Dunn’s correction for pairwise comparisons of continuous variables among HCs, AH patients, and HDCs. Mann Whitney test for comparing AH patients versus HDCs. Chi-square test for analysis of categorical variables. ^a^Treatment status for one participant was not available. ^###^ *p* < 0.001 for comparison between AH patients and HC; *p < 0.05, ***p < 0.001 for comparison between AH patients and HDC; ^$$^ *p* < 0.01 for comparison between HDC and HC. *p* < 0.05 was considered statistically significant (bolded).

Exclusion criteria for the TREAT study included serious medical conditions such as congestive heart failure, chronic obstructive pulmonary disease (COPD), cancer, uncontrolled diabetes, and chronic renal failure. Additionally, individuals with a history of jaundice or signs of end-stage liver disease (e.g., ascites, hepatic encephalopathy, variceal bleeding), chronic HBV or HCV infection, any systemic infection within 4 weeks prior to the study, or recent major surgeries within the past 3 months were excluded (47). Those with other liver diseases, such as autoimmune liver disease, hemochromatosis, and Wilson’s disease, were also excluded.

This study was approved by the Institutional Review Boards (IRB) at Indiana University School of Medicine, Mayo Clinic, and Virginia Commonwealth University. All participants provided a written informed consent form before blood was drawn. Peripheral blood was collected in heparin-coated tubes (BD Biosciences, Franklin Lakes, NJ). Plasma was prepared within 2 hours of blood collection and stored at -80°C until use. Plasma samples from 35 age- and sex -matched healthy volunteers were included as HCs.

### Liquid chromatography with tandem mass spectrometry

Plasma samples from 10 AH patients, 10 HDCs, and 10 HCs were subjected to LC-MS/MS analysis for the quantitative assessment of PLMs and SPMs. This analytic work was conducted at the Center for Salivary Diagnostics, the Forsyth Institute, Harvard School of Dental Medicine (Cambridge, MA) (48). Briefly, each plasma sample (1 mL) was mixed with internal labeled standards such as d8-5S-HETE, d4-LTB4, d5-LXA4, d5-RvD2, and d4-PGE2 in ice-cold methanol to facilitate the calculation of quantification and sample recovery (48). The mixtures were subjected to solid phase extraction using C18 cartridges. Extracts were dried using the automated evaporate system (TurboVap, Charlotte, NC), and immediately used for LC-MS/MS automated injections. The LC-MS-MS system, a Shimadzu LC-20AD HPLC and a Shimadzu SIL-20AC autoinjector (Shimadzu, Kyoto, Japan), paired with a QTrap 6500 (ABSciex, Framingham, MA), were employed to process all plasma samples. PLMs, SPM intermediates, and SPMs were identified in accordance with published criteria (17, 48, 49), including matching retention time (RT) and at least six characteristic and diagnostic ions (48). Quantitation was carried out using linear regression compared with standard curves from the synthetic and authentic solvents. LC-MS/MS data analysis was performed on the Sciex software platform, Analyst version 1.6 (Sciex, Framingham, MA) (50).

### Enzyme-linked immunosorbent assay and multiplex immunoassay

Plasma levels of PGD2, PGE2, LTB4, LXA4, RvE1, RvD2, and MaR1 were quantified using the Prostaglandin D2 ELISA Kit (Cayman Chemical, Ann Arbor, MI), Prostaglandin E2 ELISA Kit (Cayman Chemical, Ann Arbor, MI), LTB4 Parameter Assay Kit (R&D Systems, Minneapolis, MN), Lipoxin A4 ELISA Kit (Neogen, Lexington, KY), Human Resolvin E1 ELISA Kit (MBS025958, MyBioSource, San Diego, CA), Resolvin D2 ELISA Kit (Cayman Chemical, Ann Arbor, MI), and Maresin 1 ELISA Kit (Cayman Chemical, Ann Arbor, MI), respectively. Plasma levels of the systematic inflammation marker CRP and the bacterial translocation markers, including LPS binding protein (LBP), soluble CD14 (sCD14), and soluble CD163, were measured using the Human C-Reactive Protein/CRP DuoSet ELISA Kit, Human LBP DuoSet ELISA Kit, Human CD14 Quantikine ELISA Kit, and Human CD163 Quantikine ELISA Kit, respectively. These ELISA kits were purchased from R&D Systems (Minneapolis, MN).

Plasma levels of 45 inflammatory mediators, including 26 cytokines (GM-CSF, IFN-α, IFN-γ, IL-1α, IL-1β, IL-1RA, IL-2, IL-4, IL-5, IL-6, IL-7, IL-8, IL-9, IL-10, IL-12p70, IL-13, IL-15, IL-17A, IL-18, IL-21, IL-22, IL-23, IL-27, IL-31, TNF-α, and TNF-β), 8 chemokines (Eotaxin (CCL11), GRO-α (CXCL1), IP-10 (CXCL10), MCP-1 (CCL2), MIP-1α (CCL3), MIP-1β (CCL4), RANTES (CCL5), and SDF-1α), and 11 growth factors (BDNF, EGF, FGF-2, HGF, LIF, NGF-β, PDGF-BB, PlGF-1, SCF, VEGF-A, and VEGF-D) were simultaneously quantified using the Cytokine/Chemokine/Growth Factor 45-Plex Human ProcartaPlex Panel 1 (EPX450-12171-901, Invitrogen, Waltham, MA), as previously described (51). The concentrations of these cytokines/chemokines/growth factors were calculated using the Bio-Plex Manager v6.1 software (Bio-Rad, Hercules, CA). For statistical analyses, values below the detection limit of the assay were replaced with the minimal detectable concentrations for each analyte as provided by the manufacturer.

### Data mining of public RNA-seq datasets of liver tissue, monocytes, and neutrophils from AH patients and HCs

Data mining was performed to examine differential expression of genes involved in production of LMs in liver tissue from AH patients and HCs. RNA expression levels were pooled from 3 liver tissue RNA-seq databases GSE143318 (AH, n=5; HC, n=5), GSE142530 (AH, n=10; HC, n=12), and GSE155907 (AH, n=5; HC, n=4). RNA expression of genes involved in LM production in peripheral blood CD14^+^ monocytes from patients with severe AH (n=4) and HCs (n=6) was obtained from the RNA-seq dataset GSE135285. RNA expression levels were also extracted from the neutrophil RNA-seq database GSE1710809 to assess differential expression of genes involved in production of LMs in peripheral blood neutrophils in AH patients (n=3) and HCs (n=3). Data are presented as transcripts per kilobase million (TPM) normalized expression values.

### Statistical analysis

Chi-square test was used for comparison between groups for categorical variables. Mann-Whitney test and Kruskal-Wallis test with Dunn’s corrections were used to calculate differences in continuous variables between 2 groups and among 3 groups in cross-sectional analysis, respectively. A two-tailed t-test was used to calculate differences in the expression of genes involved in the production of LMs between AH patients and HC. An ordinary one-way ANOVA with Holm-Sidak’s multiple comparisons test was used to compare differences in the expression of these genes involved in production of LMs among subsets of neutrophils from AH patients and HC. The linear relationship between LMs and clinical parameters or inflammatory mediators was analyzed using the Spearman correlation test or multivariate linear regression. Wilcoxon matched-pairs signed rank test was used to calculate the differences in longitudinal analysis. *p <*0.05 was considered statistically significant.

## Results

### Characteristics of the study cohort

This study cohort included 58 AH patients, 29 HDCs, and 35 HCs at baseline, as well as 13 and 9 alcohol abstinent AH patients at 6- and 12-month follow-ups, respectively. The demographic and clinical characteristics of these participants at baseline are listed in **Table 1**. There were no significant differences in age, gender distributions, or creatine levels among AH patients, HDCs, and HCs. Compared to HDCs and HCs, AH patients had elevated levels of total bilirubin, alanine transaminase (ALT), and aspartate aminotransferase (AST). HDCs and HCs had comparable levels of total bilirubin and AST, but HDCs had higher levels of ALT than HCs. AH patients had longer prothrombin time and higher Model for End-Stage Liver Disease (MELD) score than HDCs. AH patients had more peripheral blood neutrophils but fewer platelets than HDC. AH patients also had significantly lower body mass index (BMI) than HDC. Approximately 60% AH patients were treated with prednisone at enrollment. Among the 58 AH patients at baseline, a total of 9 died within 12 months after enrollment. The 30-day mortality, 6-month, and 12-month mortality were 6.9% (n=4), 13.8% (n=8), and 15.5% (n=9), respectively. AH patients and HDCs reported similar drinking patterns (total drinking days and average number of drinks per day) over the preceding 30 days. Characteristics of the AH patients who achieved complete alcohol abstinence for 6-month or 12-month intervals as compared to HCs are shown in **Supplementary Table S1**. There were no differences in age, gender distribution, and creatinine levels between HCs and AH patients at baseline or follow-ups. Compared to HCs, AH patients at baseline and 6-month follow-up had increased levels of total bilirubin, ALT, and AST. At 12-month follow-up, most of those liver biochemistries in the AH subjects were normalized, except for ALT level, which still exhibited a trending increase (*p* = 0.065). For the AH subjects, both the prothrombin time and MELD score showed significant improvement at either the 6- or 12-follow-up in comparison to the baseline values. Due to the limited neutrophil data available for the follow-up samples, we were unable to evaluate their longitudinal changes. However, the follow-up platelet counts were consistent with the baseline values.

### AH patients had impaired biosynthetic switch from PLMs to SPMs

To elucidate the distinctions in the profiles of circulating PLMs and SPMs between AH patients and HDCs as well as HCs, we first performed LC-MS/MS to quantify plasma levels of LMs and their precursors. LC-MS/MS offers several analytical advantages, including an extended linear dynamic range, the capability to simultaneously quantify multiple metabolic analytes, high accuracy and precision due to the use of internal standards, and the avoidance of the necessity for immunological reagents (52). In our study, LC-MS/MS detected 18 LM species, including ω-3 PUFA metabolites (14-HDHA, 17-HDHA, and 18-HEPE), arachidonic acid (AA) metabolites such as leukotrienes (LTB4) and prostaglandins (PGD2 and PGE2), and SPMs such as lipoxins (LXA4 and LXB4), maresins (Mar1 and Mar2), protectins (PD1 and PDX), and resolvins (RvD1, RvD2, RvD3, RvD4, RvD5, and RvE1) (**Supplementary Table S2**). Notably, only 8 LMs were consistently detected in all 30 plasma samples, including LTB4, PGD2, PGE2, the RvE precursor 18-HEPE, RvD1/RvD2 and their precursor 17-HDHA, and MaR precursor 14-HDHA. The remaining 10 LMs were detected in only a small subset of the 30 samples, ranging from 0-10. Those 10 LMs were not further analyzed. We also quantified levels of 3 PLMs (LTB4, PGD2, and PGE2) and 4 SPMs (LXA4, RvE1, RvD2, and MaR1) in a larger set of plasma samples using ELISAs. We grouped the comparisons of the LM levels among the AH patients, HDCs, and HCs according to the AA, EPA, and DHA metabolic pathways.

**Figure 1:**
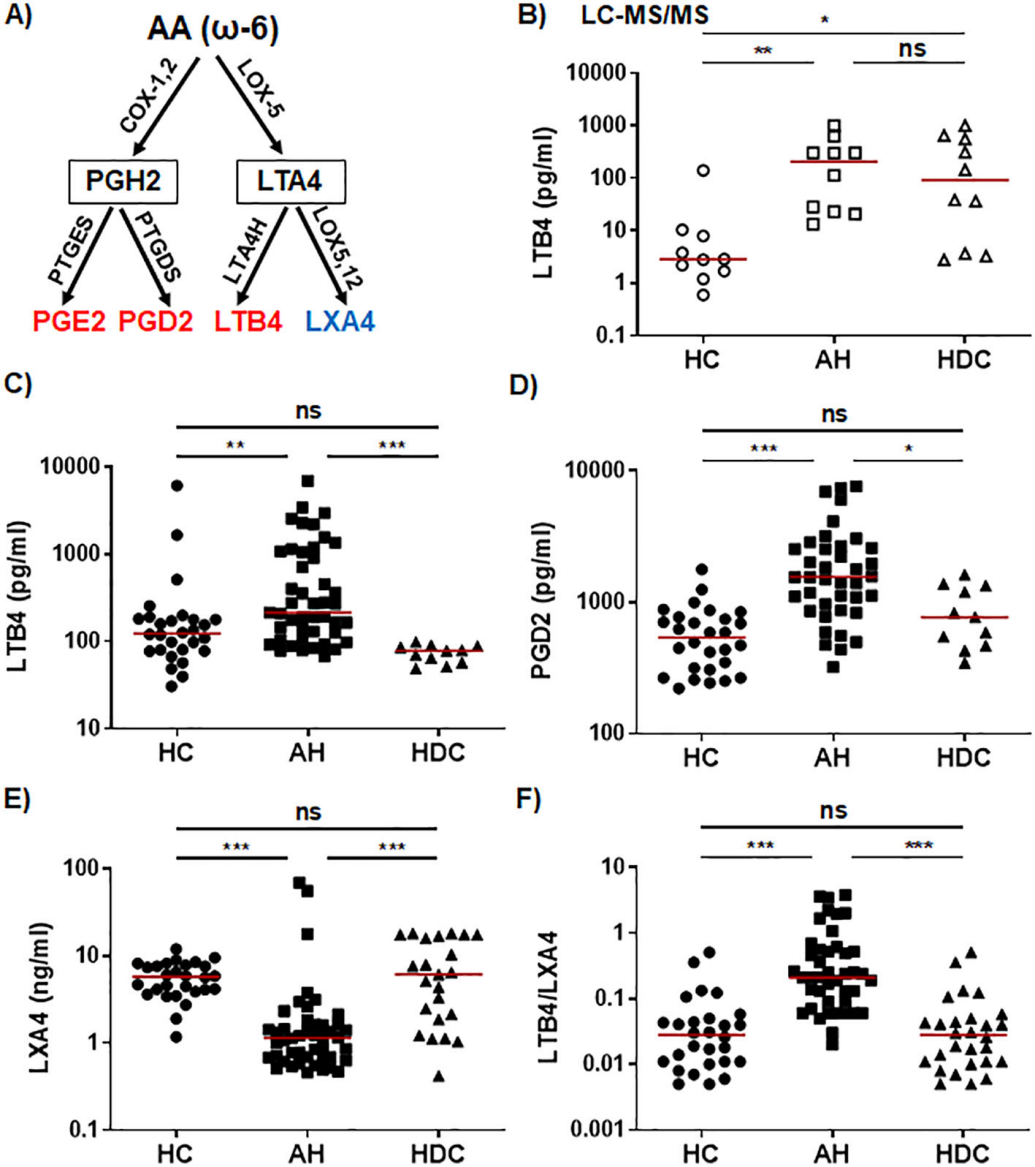
Dysregulated lipid mediators from the AA pathway in AH patients. **(A)** Simplified schematic representation of the AA metabolic pathway. Key biosynthetic enzymes are shown next to arrows, lipid intermediators in box, and proinflammatory lipid mediators in red and anti-inflammatory lipid in blue. **(B–F)** Scatter plots showing plasma levels of LTB4 **(B, C)**, PGD2 **(D)**, LXA4 **(E)**, and LTB4/LXA4 ratio **(F)** in healthy controls (HC), patients with alcoholic hepatitis (AH), and heavy drinking controls (HDC). Concentrations were measured by LC-MS/MS (B; open symbols) or ELISA (C-E; filled symbols). Kruskal-Wallis test with Dunn’s correction for pairwise comparison among AH, HDC, and HC. *p < 0.05, **p < 0.01, ***p < 0.001. ns, not significant.

In the ω-6 AA pathway (**Figure 1A**), the LC-MS/MS results revealed that AH patients had markedly higher plasma levels of the proinflammatory LTB4 (**Figure 1B**), which was corroborated by the ELISA results (**Figure 1C**). LC-MS/MS, but not ELISA, detected a higher LTB4 level in HDCs when compared with HCs (**Figures 1B, C**). The PLM PGD2 levels were notably elevated in AH patients when compared to HCs and HDCs by ELISA, whereas HDCs had similar levels of PGD2 to HCs (**Figure 1D**). By LC-MS/MS, PGD2 levels in AH patients trended higher compared to HDC (p = 0.08; **Supplementary Figure S1A**) The levels of the PGE2 were not significantly different among AH patients, HDCs, and HCs by LC-MS/MS or ELISA (**Supplementary Figures S1B, S1C**). Two SPMs (LXA4 and LXB4) in the AA pathway were detected in only a subset of plasma samples by LC-MS/MS (2 for LXA4 and 8 for LXB4) (**Supplementary Table S2**). However, ELISA consistently detected LXA4 in all plasma samples tested, revealing that LXA4 levels were significantly reduced in AH patients compared to either HCs or HDCs (**Figure 1E**). As the proinflammatory LTB4 and the anti-inflammatory LXA4 are derived from a common precursor LTA4, we also compared the LTB4/LXA4 ratio among AH patients, HDCs, and HCs. In line with a higher level of LTB4 and a reduced level of LXA4 in AH patients, the LTB4/LXA4 ratio was highly elevated in AH patients relative to HDCs and HCs (**Figure 1F**). No difference in LXA4 or LTB4/LXA4 ratio was found between HDCs and HCs (**Figures 1E, F**).

**Figure 2:**
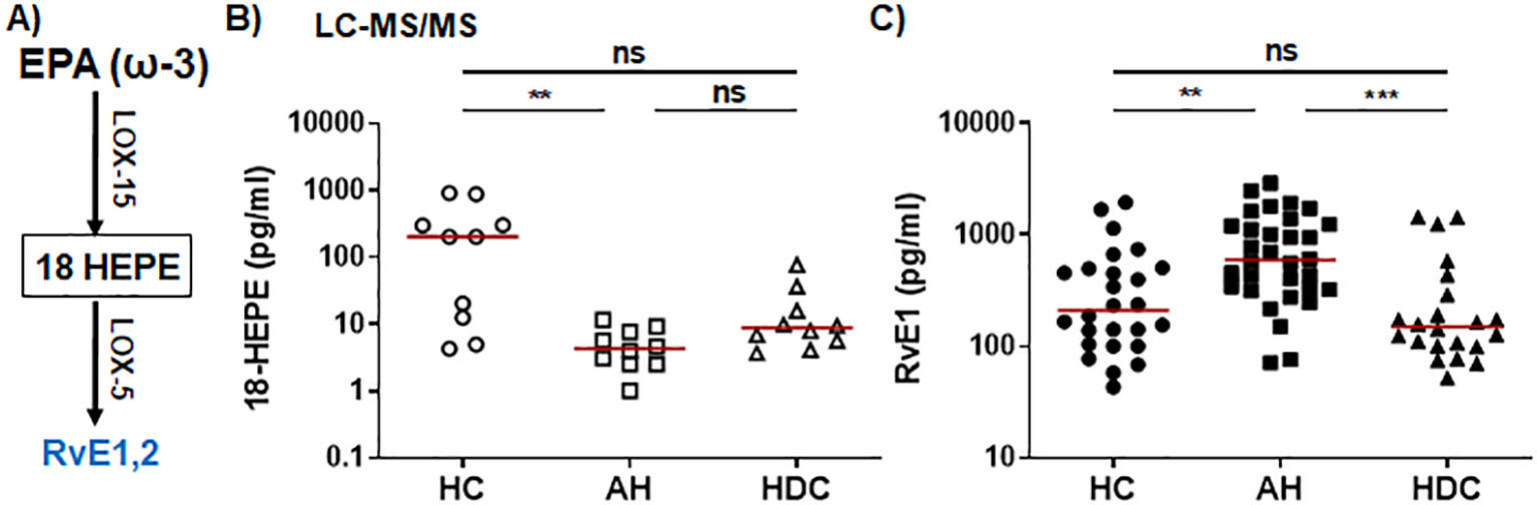
Dysregulated RVE1 and its precursor 18-HEPE from the EPA pathway in AH patients. **(A)** Simplified schematic representation of the EPA metabolic pathway. Key biosynthetic enzymes are shown next to arrows, lipid intermediator in box, and anti-inflammatory SPMs in blue. **(B, C)** Scatter plots showing plasma levels of 18-HEPE **(B)** and RVE1 **(C)** in healthy controls (HC), patients with alcoholic hepatitis (AH), and heavy drinking controls (HDC). Concentrations were measured by LC-MS/MS (B; open symbols) or ELISA (C; filled symbols). Kruskal-Wallis test with Dunn’s correction for pairwise comparison among AH, HDC, and HC. **p < 0.01, ***p < 0.001. ns, not significant.

**Figure 3:**
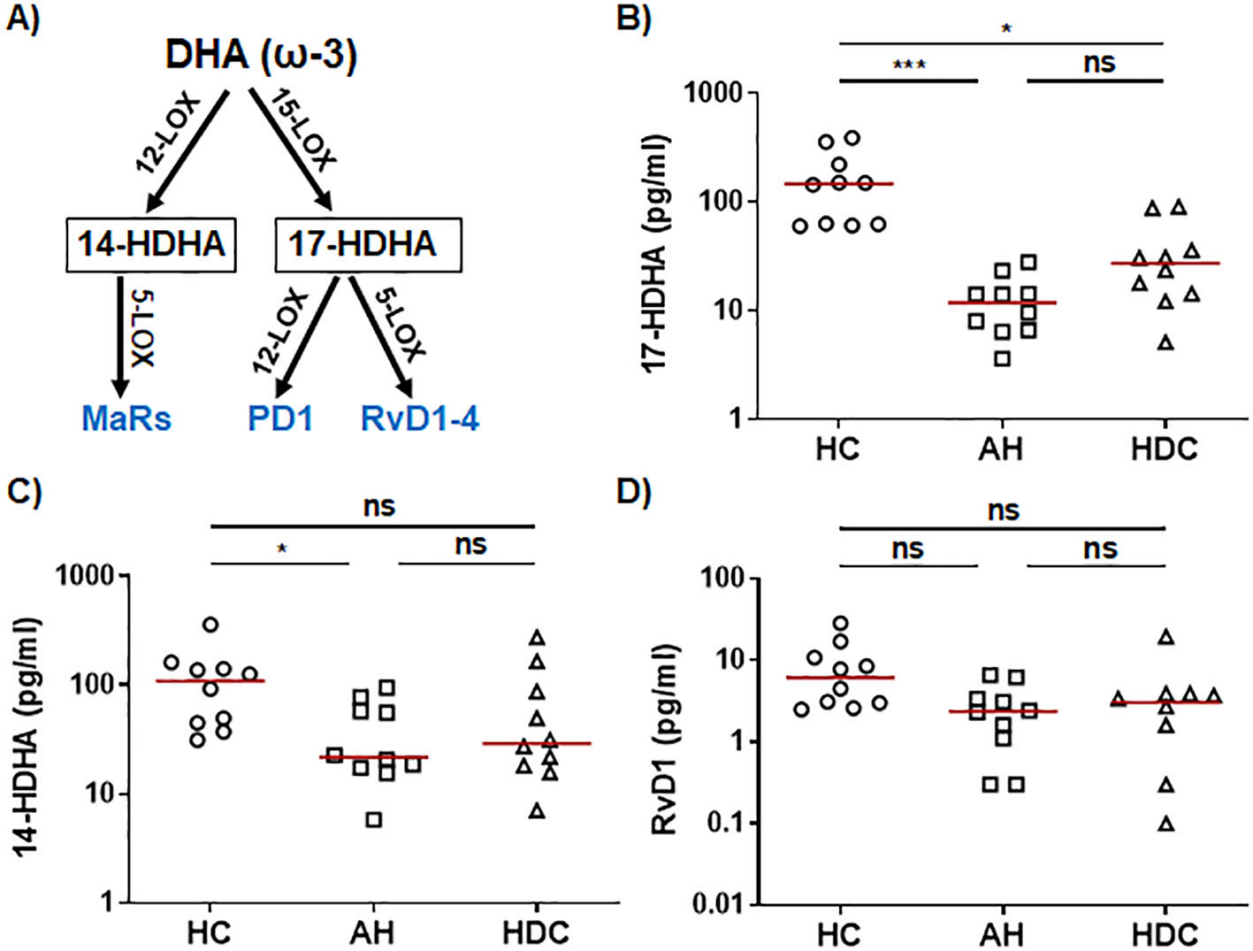
Dysregulated precursors of RvD/PD and MaR from the DHA pathway in AH patients. **(A)** Simplified schematic representation of the DHA metabolic pathway. Key biosynthetic enzymes are shown next to arrows, lipid intermediator in box, and anti-inflammatory SPMs in blue. **(B–D)** Scatter plots showing LS-MS/MS quantification of plasma levels of RvD/PD precursor 17-HDHA **(B)**, MaR precursor 14-HDHA **(C)**, and RvD1 **(D)** in healthy controls (HC), patients with alcoholic hepatitis (AH), and heavy drinking controls (HDC) (open symbols). Kruskal-Wallis test with Dunn’s correction for pairwise comparison among AH, HDC, and HC. *p < 0.05, ***p < 0.001. ns, not significant.

The ω-3 SPMs, including Rvs, protectins, and MaRs, are derived from ω-3 PUFAs such as EPA and DHA through a series of enzymatic reactions involving 5-/12-/15-LOX. In the ω-3 EPA pathway (**Figure 2A**), plasma levels of the RvE intermediate 18-HEPE were significantly lower in AH patients compared to HCs as detected by LC-MS/MS (**Figure 2B**). RvE1 was barely detectable by LC-MS/MS (**Supplementary Table S2**). However, ELISA was able to detect RvE1 in all plasma samples, showing that AH patients had significantly higher levels than HCs or HDCs (**Figure 2C**). In the ω-3 DHA pathway (**Figure 3A**), LC-MS/MS was able to detect the precursors to RvD, protectins, and MaRs (17-HDHA and 14-HDHA). Both precursors were significantly decreased in AH patients compared to HCs (**Figures 3B, C**). In addition, HDCs also had lower levels of 17-HDHA levels than HCs (**Figure 3B**). LC-MS/MS was able to detect RvD1 and RvD2 in all samples (**Supplementary Table S2**). There were no significant differences in the levels of RvD1 and RvD2 among AH patients, HDCs, and HCs (**Figure 3D**, **Supplementary Figures S1D, S1E**). Of note, RvD1 levels in AH patients displayed a trend towards lower values compared to HCs (*p* = 0.066). MaR1 and MaR2 were not detected in most samples by LC-MS/MS. MaR1 was detectable by ELISA, but no discernible changes were observed in AH patients compared to HDCs and HCs (**Supplementary Figure S1F**). Taken together, the results from our LC-MS/MS and ELISA analyses collectively suggest a pronounced imbalance in LM production in AH patients. This imbalance appears to skew towards elevated levels of PLMs and reduced levels of anti-inflammatory SPMs and their precursors when compared to HDCs and HCs.

Given that women appear to have an increased risk of developing severe AH, we examined gender differences in plasma levels of several key PLMs (LTB4, PGD2, and PGE2) and SPMs (LXA4, MaR1, RvD2, and RvE1) in AH patients. As shown in **Supplementary Table S3**, no significant differences were observed between males and females for any of the LMs tested. Additionally, the levels of these PLMs and SPMs did not differ significantly between AH patients treated with corticosteroids (**Supplementary Table S4**). Furthermore, there were no significant differences in these LM levels between AH patients who survived 12 months post-enrollment and those who deceased (**Supplementary Table S5**).

### Alcohol abstinence reversed dysregulated production of LMs

**Figure 4:**
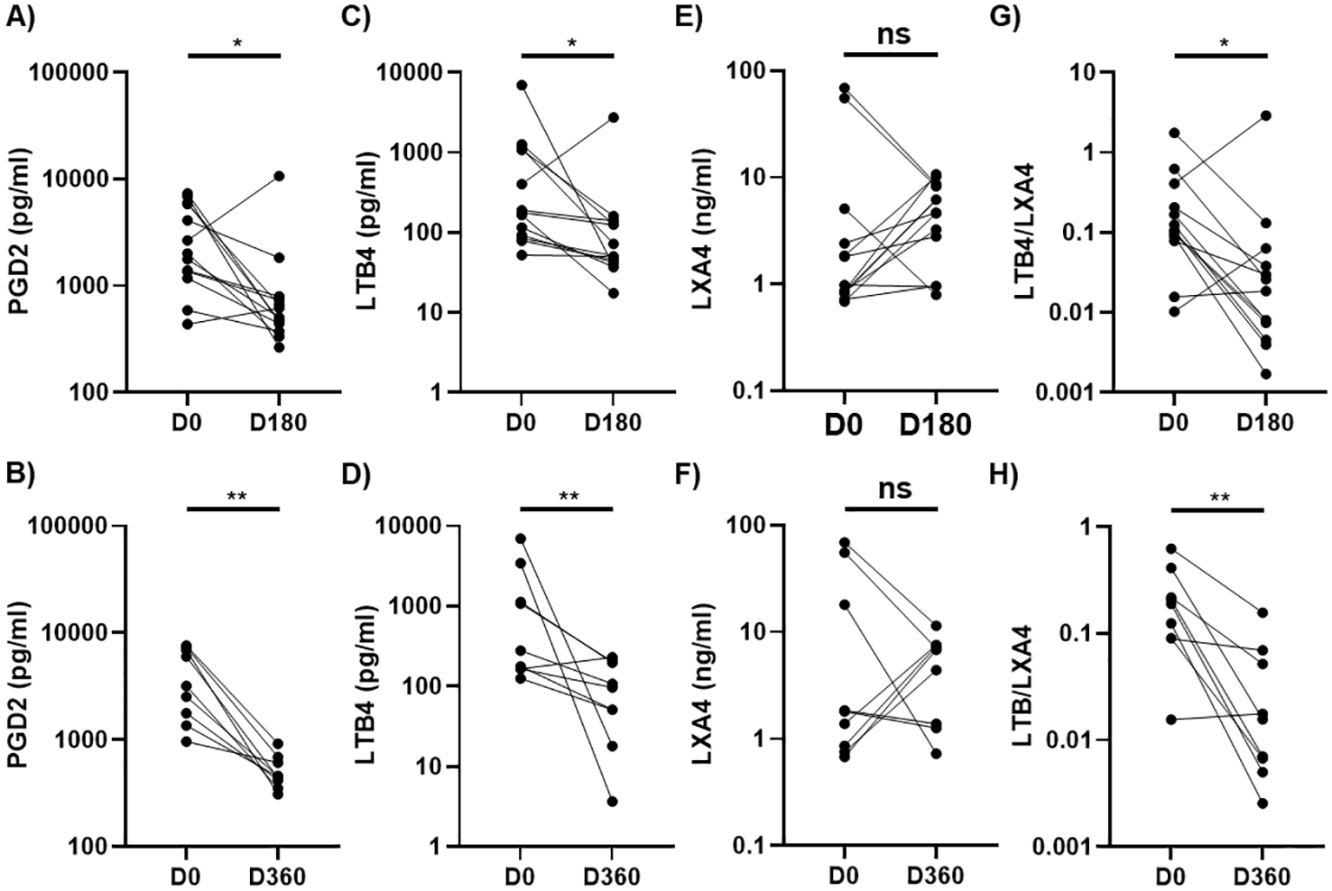
Longitudinal changes in plasma levels of PGD2, LTB4, and LXA4 in AH patients at 6-month or 12-month follow-ups. Scatter plots comparing levels of PGD2 **(A, B)**, LTB4 **(C, D)**, LXA4 **(E, F)**, and LTB4/LXA4 ratio **(G, H)** at 6-month (D180) or 12-month (D360) follow-ups with baseline values (D0) in patients with alcoholic hepatitis (AH) who were abstinent during the follow ups. Wilcoxon matched pairs signed rank test was used to compare the differences between D0 and D180 (n=13) or D360 (n=9). *p < 0.05, **p < 0.01. ns, not significant. AH, alcoholic hepatitis.

Next, we conducted a longitudinal assessment to evaluate changes in three highly dysregulated LMs (PGD2, LTB4, LXA4) and the LTB4/LXA4 ratio in AH patients who achieved complete abstinence at 6-month (n=13) and 12-month (n=9) follow-ups, with 5 AH patients providing paired samples. Compared to baseline values, PGD2 and LTB4 levels, as well as the LTB4/LXA4 ratio, were significantly reduced (**Figures 4A–D, G, H**), while LXA4 levels remained relatively stable with no significant changes observed at either follow-up (**Figures 4E, F**). Additionally, we assessed whether these lipid mediator levels normalized during follow-up. At follow-ups, elevated baseline levels of PGD2, LTB4, and the LTB4/LXA4 ratio had regressed to levels comparable to those in HCs, while the initially reduced levels of LXA4 increased to levels analogous to those in HCs (**Supplementary Figure S2**). Thus, the levels of PGD2, LTB4, LXA4, and the LTB4/LXA4 ratio were normalized in abstinent AH patients over the course of the 6- and 12-month follow-ups.

### Data mining for gene expression of critical enzymes involved in LM production in AH patients

To unravel the underlying mechanisms contributing to the dysregulated production of LMs in AH patients, we conducted a comprehensive analysis of three publicly available RNA-seq databases (GSE143318, GSE142530, GSE155907). Our objective was to assess the differential expression of genes that encode several critical enzymes involved in LM production within liver tissue from AH patients and HCs serving as liver donors. The combined dataset encompassed RNA sequences from 20 AH patients and 21 HCs. We observed that the expression of COX-1 or COX-2, which converts the ω-6 AA PUFA to the precursor of PGE2 and PGD2 (PGH2), was not different between AH patients and HCs (**Supplementary Figures S3A, S3B**). However, expression of the downstream PGD2 synthetase (PTGDS) and PGE2 synthetase (PTGES) were significantly upregulated in the liver tissue from AH patients (**Supplementary Figures S3C, S3D**). In addition, the gene encoding the enzyme LTA4 dehydrogenase (LTA4H), responsible for converting LTA4 into LTB4 was also elevated in AH patients (**Supplementary Figure S3E**). The genes encoding for 5-LOX, an enzyme involved in the production of both PLMs (LTB4) and SPMs in the AA (LXA4 and LXB4) and the ω-3 pathways, were highly upregulated in the liver tissue from the AH patients (**Supplementary Figure S3F**). The gene encoding for 12-LOX that is essential for generating SPMs from both ω-6 and ω-3 pathways were also significantly enhanced in the liver tissue of AH patients (**Supplementary Figure S3G**). On the other hand, expression of 15-LOX, encoding for another critical LOX involved in SPM production, exhibited a trend towards higher in AH patients compared to HCs (*p* = 0.07). Together, liver tissue from AH patients exhibits a distinct gene expression profile indicative of heightened production of LMs, especially PLMs.

Monocytes/macrophages and neutrophils play central roles in inflammation initiation and resolution by producing a plethora of proinflammatory and anti-inflammatory mediators, as well as phagocytosing cellular debris and injured cells (53–55). We analyzed RNA expression of genes involved in LM production in peripheral blood monocytes (GSE135285) and neutrophils from AH patients (GSE171809). CD14^+^ monocytes from patients with severe AH (n=4) and HCs (n=6) had comparable expression levels of COX-1, COX-2, PTGES, LTA4H, 5-LOX, 12-LOX, and 15-LOX (**Supplementary Figures S4A, S4B, S4D–H**), with exception of PTGDS, which showed a higher expression trend in the monocytes from AH patients (p=0.055, **Supplementary Figure S4C**). A recent study identified a unique and functionally exhausted subpopulation of neutrophils, referred to as low-density neutrophils (LDN), in the AH subjects that are different from the conventional high-density neutrophils (HDN) (56). Interestingly, LDN from AH patients expressed notably higher levels of COX-1, COX-2, and PTGDS than HDN in AH subjects and HCs. In contrast, the expression of these genes in HDN form AH subjects and HCs were comparable (**Supplementary Figures S5A–C**). LTA4H levels were also higher in LDN than HDN (**Supplementary Figure S5E**), whereas levels of PTGES (**Supplementary Figure S5D**) and 15-LOX PTGES (**Supplementary Figure S5H**) were not different between the two subpopulations of neutrophils. Both 5-LOX and 12-LOX in LDN were downregulated in AH subjects compared to that from HCs. In addition, 12-LOX expression in HDN from AH subjects was also lower than those from HCs. However, 5-LOX levels in HDN were higher than both LDN and neutrophils from HCs (**Supplementary Figures S5F, S5G**). These data suggest that peripheral LDNs in AH patients possess the potential to produce higher levels of PLMs while generating lower levels of SPMs.

### Correlation of plasma levels of LMs with disease severity and inflammatory mediators in AH patients

To examine whether LMs might be linked to the pathogenesis of AH, we performed Spearman correlation analyses to assess their associations with age, AH disease severity markers, including creatinine, liver-related biochemical measurements (total bilirubin, AST, and ALT), prothrombin time, and MELD score, neutrophil and platelet counts, and BMI in AH patients. Plasma levels of PGD2, PDE2, LTB4, LXA4, MaR1, and RvE1 as determined by ELISA were used for these analyses. We also analyzed their correlations with the LTB4/LXA4 ratio. The results of these correlations are summarized in **Table 2**. Age was not associated with plasma levels of these LMs except having a positive correlation with LTB4. BMI also did not correlate with any of the LMs tested. Upregulated PGD2 positively correlated with total bilirubin. MaR1 exhibited a negative correlation with ALT and a positive correlation with platelets. LTB4 and LXA4, as well as PGE2 and RvE1, did not significantly correlate with any of these markers of disease severity. Notably, the elevated LTB4/LXA4 ratio displayed a positive correlation with both the total bilirubin levels and the MELD score (**Table 2**), suggesting this specific ratio may hold potential as an indicator of disease severity in AH patients.

**Table 2:** Correlations of altered lipid mediators with clinical parameters and inflammatory markers in patients with alcoholic hepatitis.

Categories	Variables	PGD2	PGE2	LTB4	LXA4	LTB4/LXA4	RvE1	MaR1
**Demo-graphics**	Age	0.17	-0.22	0.33*	0.21	0.08	0.28	-0.07
**Clinical parameters**	Creatinine	0.27	-0.11	0.05	-0.10	0.16	0.24	-0.23
	Total Bilirubin	0.42**	0.10	0.14	-0.22	0.32*	0.16	0.04
	ALT	0.08	-0.25	0.06	-0.20	0.11	-0.01	-0.36*
	AST	0.25	-0.19	0.05	-0.13	-0.10	0.06	-0.16
	INR	-0.17	-0.37	0.10	0.18	0.09	0.07	-0.26
	MELD	0.25	0.02	0.13	-0.19	0.31*	0.16	-0.14
	Neutrophils	0.17	-0.16	0.19	-0.22	0.24	-0.03	-0.21
	Platelets	0.28	0.35	-0.10	-0.14	-0.07	-0.33	0.46**
	BMI	0.02	-0.20	0.05	-0.01	0.12	0.21	0.13
**Inflammation markers**	CRP	0.55*	0.47	0.25	-0.13	0.37	-0.32	0.57
	LPS	0.16	0.14	0.08	0.22	-0.07	-0.19	0.12
	LBP	0.13	0.14	-0.06	-0.43**	0.16	-0.33	-0.04
	sCD14	0.06	-0.11	0.04	-0.50**	0.28	0.29	0.28
	sCD163	0.15	0.07	0.02	-0.14	0.24	0.38	0.23
**Cytokines**	IFN-a	0.23	0.78*	0.02	-0.05	0.07	N/A	N/A
	IL-1RA	0.35	0.27	0.38	-0.38	0.55**	N/A	N/A
	IL-8	0.15	-0.62	0.31	-0.34	0.47*	N/A	N/A
	IL-10	-0.11	0.29	-0.16	-0.45*	0.07	N/A	N/A
	IL-13	0.30	0.25	0.36	0.18	0.17	N/A	N/A
	TNF-α	0.11	0.72*	-0.02	-0.20	-0.01	N/A	N/A
**Chemokines**	IP10	0.48*	0.62	-0.10	-0.13	-0.13	N/A	N/A
	MIP-1α	0.45	0.38	0.31	0.06	0.19	N/A	N/A
	MIP-1β	0.57*	0.33	0.23	0.02	0.13	N/A	N/A
	RANTES	0.56*	0.57	0.15	0.14	0.10	N/A	N/A
**Growth factors**	FGF-2	-0.01	0.55	0.13	0.46*	-0.06	N/A	N/A
	HGF	0.34	0.07	-0.15	-0.59**	0.38	N/A	N/A
	VEGF	0.52*	0.41	0.06	-0.24	0.17	N/A	N/A

PGD2, prostaglandin D2**;** PGE2, prostaglandin E2; LTB4, leukotriene B4; LXA4, lipoxin A4; MaR1, maresin 1; ALT, alanine aminotransferase; AST, aspartate aminotransferase; INR, international normalized ratio; MELD, model for end-stage liver disease; BMI, body mass index; CRP, C-reactive protein; LPS, lipopolysaccharides; LBP, lipopolysaccharides binding protein; sCD14, soluble CD14; sCD163, soluble CD163; N/A, not analyzed due to insufficient data points. The numbers represent Spearman’s coefficients, *p < 0.05, **p < 0.01 (bolded).

Next, we performed multivariable linear regression analysis, adjusting for age due to its association with LTB4. We selected three lipid mediators, PGD2, LTB4/LXA4, and MaR1 that showed significant correlations with clinical parameters in the Spearman correlation analysis, for the multivariate analysis. The model included 13 samples with complete data for these lipid mediators, age, and 8 clinical parameters. Given the limited number of observations, we used several separate multiple regressions rather than a single multivariate multiple regression. As shown in **Table 3**, elevated PGD2 was positively correlated with several clinical parameters, including creatinine, AST, MELD score, and total bilirubin. The LTB4/LXA4 ratio was negatively correlated with creatinine and AST, while MaR1 was negatively correlated with creatinine.

**Table 3:** Multivariate linear regression analysis of lipid mediators with clinical parameters in patients with alcoholic hepatitis.

Variables	Prostaglandin D2			Leukotriene B4/lipoxin A4 ratio			Maresin 1		
	*Beta*	95% CI	*p*	*Beta*	95% CI	*p*	*Beta*	95% CI	*p*
Creatinine	0.002	0.002, 0.003	**<0.001**	-1.394	-2.009, -0.780	**0.001**	-0.014	-0.021, -0.008	**0.001**
Total Bilirubin	0.013	0.0034, 0.022	**0.014**	0.795	-7.989, 9.579	0.840	-0.026	-0.122, 0.071	0.556
ALT	0.002	-0.026, 0.030	0.882	14.990	-11.417, 41.397	0.227	0.051	-0.239, 0.341	0.694
AST	0.078	0.018, 0.138	**0.017**	-60.339	-116.763, -3.9147	**0.039**	-0.132	-0.752, 0.487	0.635
INR	-0.0002	-0.001, 0.0004	0.454	0.043	-0.570, 0.654	0.875	0.0001	-0.007, 0.007	0.961
MELD	0.007	0.001, 0.014	**0.048**	-3.184	-0.977, 3.406	0.298	-0.045	-0.117, 0.028	0.191
Neutrophils	-0.002	-0.008, 0.004	0.441	3.02	-2.831, 8.870	0.584	-0.016	-0.080, 0.048	0.268
Platelets	0.031	-0.044, 0.105	0.369	-6.495	-76.533, 63.543	0.836	0.276	-0.493, 1.045	0.432

CI, confidence interval; ALT, alanine aminotransferase; AST, aspartate aminotransferase; INR, international normalized ratio; MELD, model for end-stage liver disease. Multivariate linear regression analyses were performed with age as a cofounding factor. The numbers represent correlation coefficients (*Beta*), 95% CI, and *p* value (*p*). *p* < 0.05 (bolded) was considered significant.

AH patients are in a hyperinflammatory state, primarily driven by alcohol-induced MT (8, 9, 12, 13). Inflammation and MT play pivotal roles in the development and progression of AH and also regulate the biosynthesis of LMs (57–59). We first analyzed the associations of the 6 LMs (PGD2, PGE2, LTB4, LXA4, MaR1, and RvE1) and the LTB4/LXA4 ratio with upregulated markers of systemic inflammation (CRP) and MT (LPS, LBP, sCD14, and sCD163) (10, 11) (**Supplementary Table S5**). The correlations were summarized in **Table 2**. PGD2 had a significant positive correlation with plasma levels of CRP, which were highly elevated in AH patients as compared to both HDC and HC (**Supplementary Table S5**), whereas LXA4 correlated negatively with LBP and sCD14. None of the 6 LMs correlated with circulatory levels of LPS or sCD163 (**Table 2**). Furthermore, we performed a correlation analysis between plasma levels of these LMs and inflammatory cytokines, chemokines, and growth factors as quantified through a 45-plex immunoassay. Upregulated PGD2 levels positively correlated with 3 chemokines (IP10, MIP-1β, and RANTES) and the growth factor VEGF and had a clear trend to correlate with another chemokine MIP-1a (*p* = 0.051). PGE2 showed positive correlations with the proinflammatory cytokines IFN-α and TNF-α. Downregulated LXA4 negatively correlated with the anti-inflammatory cytokine IL-10 and the growth factor HGF (hepatocyte growth factor), but positively correlated with the growth factor FGF-2. The elevated LTB4/LXA4 ratio positively correlated with IL-1RA and IL-8. Of note, plasma levels of all those associated inflammatory cytokines, chemokines, and growth factors except FGF-2, were highly upregulated in AH patients compared to HC and/or HDC (**Supplementary Table S6**). These results suggest that dysregulated production of LMs is intricately linked with dysregulated production of inflammatory cytokines, chemokines, and growth factors, and potentially contributing to the pathogenesis of AH.

## Discussion

AH is a severe and progressive liver and systemic inflammatory disease. Our recent investigations, consisting of cross-sectional and longitudinal studies within a comprehensive multicenter project (TREAT, U01AA021840), have unveiled crucial insights into the inflammatory responses, microbial translocation, the activation of immune cells and endothelial cells (ECs), and intestinal epithelium damage (4, 10, 11, 51). Our results reveal that even after 12 months of abstinence from alcohol, AH patients continue to exhibit significantly elevated levels of inflammatory mediators, such as the proinflammatory cytokines IL-8, TNF-α, IL-18, and IL-23^4^. These results strongly imply that alcohol not only triggers inflammation but also impairs the resolution of inflammation. However, little research has been conducted to study the biological events that regulate the resolution of inflammation in ALD such as AH. In this study, we used LS-MS/MS and ELISA to profile various LMs in the peripheral blood, encompassing both PLMs and SPMs, in AH patients, HDCs, and HCs. Th primary objective was to understand the biological processes orchestrating the resolution of inflammation and the restoration of normal metabolism and tissue homeostasis in AH patients. We found that AH patients had higher circulating levels of two PLMs (PGD2 and LTB4) and the SPM RvE1 compared to HDCs and/or HCs. In contrast, the SPMs LXA4, along with the precursors to RvEs (18-HEPE), RvDs (17-HDHA), and MaRs (14-HDHA) within the ω-3 pathway, were significantly reduced in AH subjects. Notably, the plasma LM profile in HDCs remained largely unaffected when compared to HC, except for higher levels of LTB4 and lower levels of 17-HDHA detected using LC-MS/MS. Intriguingly, some PLMs and SPMs correlated AH disease severity, clinical parameters, and several inflammatory cytokines positively and negatively, respectively. The dysregulation in LMs was reversed with alcohol abstinence at 6- and 12-month follow-ups, coincident with the normalization of clinical parameters of the longitudinal subjects.

The biosynthesis of PLMs and SPMs is tightly regulated in a spatiotemporal manner during inflammation. A failure to transition from PLM production to SPM synthesis can lead to impaired resolution of inflammation, contributing to the progression of chronic inflammatory and vascular disorders (60, 61). In individuals with alcohol use disorder (AUD) or AH, serum profiles of bioactive lipid metabolites derived from the oxidation of ω-6 and ω-3 PUFAs are profoundly altered, as determined by LC-MS/MS analysis (62). This study demonstrated that serum levels of LTB4, a key PLM in the ω-6 pathway, were significantly elevated and positively correlated with MELD score (62). Our study corroborates these findings, showing a markedly skewed production of PLMs and SPMs in the ω-6 AA pathway, with significantly elevated plasma levels of LTB4 in AH patients. Additionally, PGD2, another PLM in the ω-6 pathway, was significantly elevated in these patients. LTB4 is enzymatically generated from AA through the actions of 5-LOX and LTA4H, while PGD2 is biosynthesized from AA via COX-1 and COX-2 and the terminal synthase PTGDS. Consistent with the elevated plasma levels of PGD2 and LTB4 observed in AH patients, an analysis of three publicly available RNA-seq databases of liver tissue from AH subjects revealed significant upregulation of PTGDS and LTA4H gene expression.

Plasma levels of LXA4, a SPM in the ω-6 AA pathway, were markedly decreased in AH patients. Reduced circulatory levels of LXA4 are observed in various inflammatory diseases, such as cardiometabolic, neurological, and autoimmune conditions (63–65). LXs are produced through transcellular biosynthesis, which involves interactions between neutrophils and the epithelium or endothelium, or through neutrophil-platelet aggregation at sites of inflammation (63, 66). For instance, mucosal epithelial cells generate LM intermediates via 15-LOX, which are subsequently converted to LXA4 and LXB4 by neutrophil 5-LOX. Within the vascular lumen, neutrophils produce the LXA4 intermediate LTA4 via 5-LOX and then transfer it to platelets, which metabolize it into LXA4 and LXB4 via 12-LOX. The reduced circulating levels of LXA4 in AH patients were not consistent with the higher gene expression of both 5-LOX and 12-LOX observed in liver tissue from these patients. This discrepancy could be attributed to several factors, such as the high hepatic infiltration of LOX-expressing inflammatory leukocytes, low translation efficiency, post-translational modifications, reduced enzyme activity, subcellular localization of the enzymes, or the involvement of regulatory factors affecting LOX activity. Additionally, contributions from tissues or cells outside the liver could influence LXA4 production in circulation (67). Furthermore, data mining revealed that a subset of peripheral blood neutrophils, known as LDNs, expressed lower levels of 5-LOX and 12-LOX compared to conventional HDNs in AH patients, suggesting that these unconventional neutrophils may contribute to the reduced production of LXA4 in AH patients.

ω-3 SPMs, encompassing DHA-derived MaRs, protectins, and D-series Rvs, as well as EPA-derived E-series Rvs, play a pivotal role in regulating inflammation and restoring homeostasis. The levels of these ω-3 SPMs in circulation are frequently altered in chronic inflammatory diseases (68), underscoring their significance in determining disease outcomes. Our LS-MS/MS results revealed a significant decrease in the levels of precursors for Rvs, protectins, and MaRs of SPMS (18-HEPE, 17-HDHA, and 14-HDHA) in AH patients compared to HCs or HDCs. Notably, these SPM intermediates, 17-HDHA and 18-HEPE, have been used as markers for the activation levels of the MaR-, protectin-, and Rv-producing pathways, respectively (69–71). This reduction suggests a potential defect in SPM biosynthesis pathways in AH patients, which may contribute to impaired resolution of inflammation and disease progression.

RvE1 levels were upregulated in patients with AH despite a decrease in its precursor 18-HEPE. RvE1 is produced via transcellular biosynthesis through interactions between neutrophils and vascular endothelial cells. The RvE1 precursor 18-HEPE is secreted by the endothelial cells and then converted to RvE1 in neutrophils by 5-LOX (72). AH is associated with increased neutrophils in circulation and the liver (73). From RNA-seq data mining, we found that both the conventional peripheral blood neutrophils and the liver tissue from patients with AH expressed higher levels of 5-LOX. It is possible that 18-HEPE was more efficiently converted to RvE1 by the elevated levels of neutrophil 5-LOX in AH patients, leading to lower levels of 18-HEPE and higher levels of RvE1. Elevated circulating levels of SPMs along with proinflammatory mediators have been observed in several diseases (74), which might represent a failed attempt of the immune system to restore homeostasis. Further studies are warranted to dissect the mechanisms of the discorded production of 18-HEPE and RvE1 in AH and the role of RvE in immunopathogenesis of AH.

Inflammation plays a major role in driving development and disease progression of AH, with non-resolving inflammation associated with severe disease (75). Our study revealed an intricate link of the skewed profile of circulatory LMs with not only the dysregulated production of inflammatory cytokines, chemokines, and growth factors, but also importantly with disease severity of AH. A previous study showed that dysregulation of serum LMs in a group of 13 AH patients is associated with a range of liver histology and clinical scores, including a positive correlation between the elevated PLM LTB4 and MELD score (62). Although we did not observe a correlation between upregulated LTB4 and MELD score in the AH subjects, we did find that the LTB4/LXA4 ratio was positively correlated with MELD score. Additionally, the highly elevated plasma levels of PGD2 correlated with total bilirubin levels, an indicator of AH disease severity. PGD2 has both proinflammatory and anti-inflammatory properties depending on the tissue and context (76). PGD2 is mainly produced by mast cells, which can induce allergic inflammation (77). Mast cells are enriched in ALD liver and regulate liver disease progression (78). Therefore, it is possible that mast cells-released PGD2 could contribute to hepatic inflammation in AH. Liver resident macrophages Kupfer cells and liver sinusoidal endothelial cells can also produce PGD2, the major prostanoid formed in the liver (79). Activation of PGD2 receptor DP1 on hepatic stellate cells by DP1 agonism was shown to suppress the acute hepatic inflammatory response in ConA-induced hepatitis in mice (80). It remained to be determined whether PGD2 enhances or ameliorates pathogenesis of AH.

BMI plays a significant role in the pathophysiology of alcoholic hepatitis by influencing the degree of inflammation and immune activation (81). Overweight, defined as BMI ≥ 25 in women and ≥ 27 in men, is a recognized risk factor for more severe histological liver damage and the progression of alcoholic liver disease (82, 83). A higher BMI is linked to increased adipose tissue, which secretes pro-inflammatory cytokines like TNF-α and IL-6, thereby exacerbating systemic inflammation (81, 84). This heightened inflammation, when combined with alcohol’s toxic effects, drives excessive immune activation, leading to more severe liver damage in AH patients. Additionally, elevated BMI is associated with increased gut permeability and microbial translocation (85), further intensifying systemic inflammation and worsening liver injury. However, our analysis revealed that BMI did not significantly influence or correlate with the levels of lipid mediators in AH patients. Thus, while BMI is recognized as a contributor to inflammation and liver damage in AH, our findings suggest that its impact on lipid mediator levels may be minimal in this context.

Currently, there are no effective medical interventions available for ALD such as AH. Alcohol abstinence remains a cornerstone of treatment for ALD, which significantly improves the disease outcomes. However, it is important to note that alcohol abstinence typically does not lead to complete recovery for most ALD patients, consistent with our clinical observations. As previously reported, our investigations demonstrated that alcohol abstinent AH patients exhibited substantial improvements in clinical scores and liver function, although these improvements were not absolute (51). Despite alcohol abstinence, our previous studies involving this cohort of AH patients revealed the persistence of numerous up-regulated inflammatory factors, including proinflammatory cytokines (IL-8, IL-18, IL-23, and TNF-α), endothelial cell activation markers (sCD146, sICAM, and sVCAM), soluble immune checkpoints (sCD27, sCD40, sHVEM, and sTIM3), and the intestinal epithelium damage markers (REG3a and TFF3) (4, 10, 51, 86). None of these factors was completely normalized (4, 10, 51, 86). In our current study, we found that three highly dysregulated LMs (PGD2, LTB4, and LXA4) were mostly normalized in AH subjects who abstained from alcohol. While it remains unknown whether other LMs, especially SPMs, experience a similar restoration, our data strongly suggest that LMs may exhibit a more pronounced responsiveness to the cessation of alcohol consumption.

In this study, we used both LC-MS/MS and ELISAs to quantify plasma levels of multiple LMs (both PLMs and SPMs) in AH patients, HDC, and HC. LC-MS/MS offers greater accuracy and the ability to quantify multiple analytes simultaneously, while ELISA assays are more affordable and allow for the quantification of larger number of samples. However, both assays have limitations. Specifically, LC-MS/MS is expensive and only limited number of samples could be assayed. A few ELISAs including those for LXA4, PGE2, RvD2 are suggested to run with extracted samples to reduce possible interferences from complex matrix in plasma samples. However, due to the limited amount of plasma samples, all the LM ELISAs were performed with unextracted samples. The PGD2 and MaR1 ELISA kits are validated for quantification of those LMs in cell lysates and urine samples. Furthermore, PGD2 is unstable, readily metabolized in plasma. Thus, measure plasma levels of PGD2 without converting it to a more stable methoxamine derivative likely underestimated the amounts of PGD2 in the plasma samples. Additionally, 3 LM ELISAs, including those for PDG2, PGE2, and LXA4, have cross-reactivities with related LMs. For example, PGD2 ELISA has 94.2% and 21.6% cross-reactivities with PGF2a and PGJ2, respectively, while PGE2 ELISA has 43% and 18.7% cross-reactivities with PGE3 and PGE1, respectively. The LXA4 ELISA has 24.0% cross-reactivities with the aspirin-induced 15-EPI-LXA4. Lastly, our sample size is relatively small, especially the longitudinal samples and those used in the multivariate linear regression analysis. Despite those limitations, our study revealed an imbalance in the production of PLMs and SPMs in AH patients compared to HDC and HC.

In conclusion, our investigation unveils a substantial dysregulation of peripheral blood LMs, encompassing a broad spectrum of PLMs, SPMs, and their precursors in AH patients. Although HDCs had no overt clinical symptoms, they still exhibited some alterations in circulating levels of PLMs and SPMs. These abnormalities were largely reversed in AH subjects who underwent alcohol abstinence. Moreover, our study revealed correlations between altered LM levels and key clinical indicators of disease severity, as well as inflammatory factors in AH patients. Currently, there is no specific medical treatment available for AH (87). Corticosteroids remain the mainstay of treatment for severe AH, but they cause serious side effects, including immunosuppression (87). Thus, there is an urgent need to identify safer and more effective therapeutics. A promising approach involves harnessing the body’s natural resolution process of inflammation for therapeutic purposes. SPMs actively exert potent dual anti-inflammatory and pro-resolving effects without causing immunosuppression (42, 45, 59). Furthermore, SPMs contribute to the restoration of tissue metabolism and homeostasis. Therefore, SPMs emerge as a valuable and innovative tool for preventing, ameliorating, and treating AH and other forms of ALD. In a mouse model of AH, 12-/15-LOX deficiency exacerbated hepatic and systematic inflammation and increased liver disease severity (88). Remarkably, this pathological state could be effectively mitigated through the exogenous supplementation of LXA4 (88). Our study, demonstrating the skewed production of PLMs and SPMs in AH patients, adds further support to the development of inflammation resolution-based strategies for the prevention, amelioration, and treatment of AH.

## Data Availability

The original contributions presented in the study are included in the article/**Supplementary Material**. Further inquiries can be directed to the corresponding authors.
