# Supplementary material for: Disrupted balance between pro-inflammatory lipid mediators and anti-inflammatory specialized pro-resolving mediators is linked to hyperinflammation in patients with alcoholic hepatitis
Source: Front Immunol. 2024 Nov 21;15:1377236. doi: 10.3389/fimmu.2024.1377236 (PMC11617321; doi:10.3389/fimmu.2024.1377236)
Supplement: Supplementary file 1 [file DataSheet1.pdf]

Figure S1

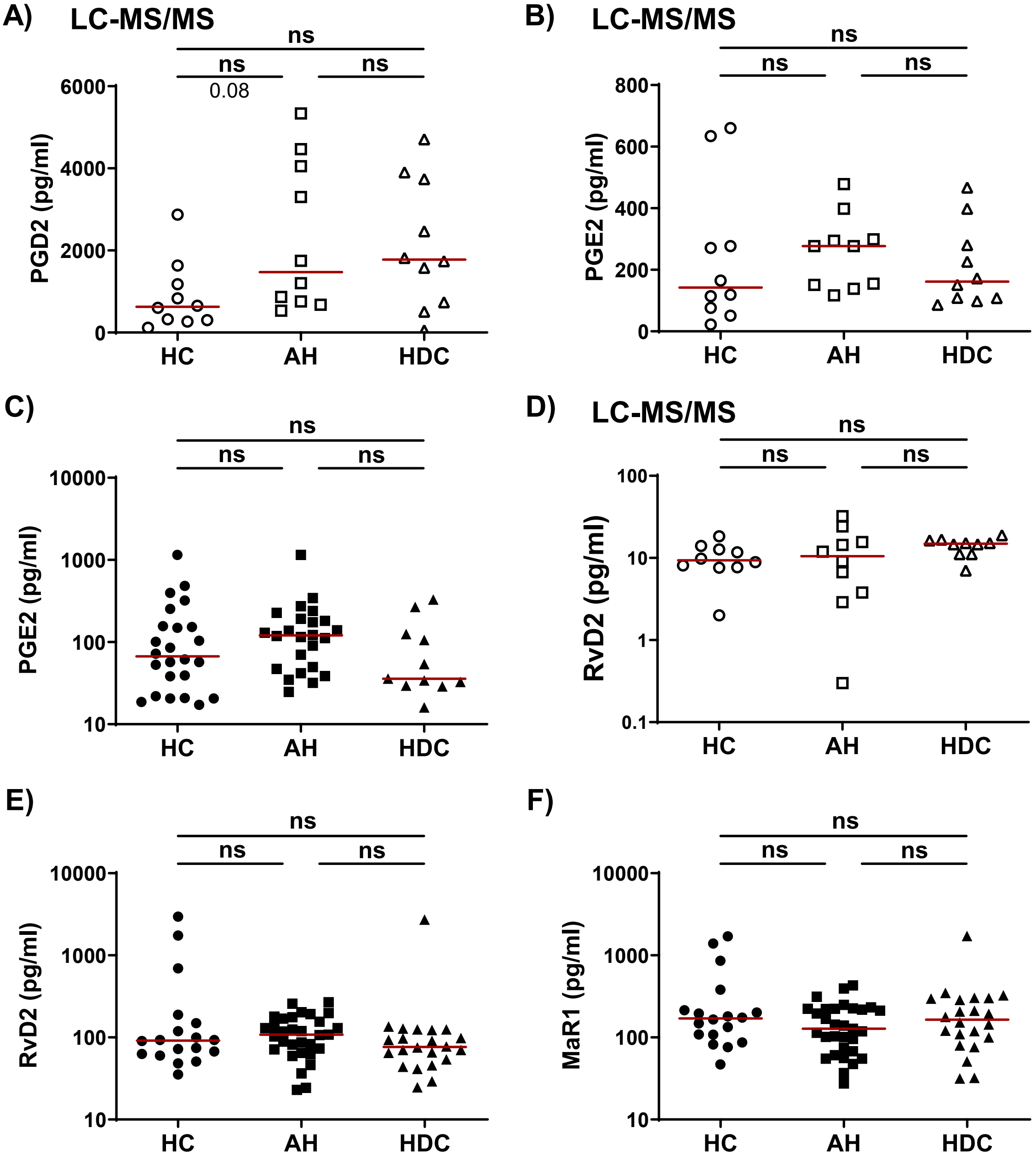

Figure S1. Scatter plots showing plasma levels of PGD2 (A), PGE2 (B, C), RvD2 (D, E), and MaR1 (F) in healthy controls (HC), patients with alcoholic hepatitis (AH), and heavy drinking controls (HDC). Kruskal-Wallis test with Dunn's correction for pairwise comparison among AH, HDC, and HC. ns, not significant.

Figure S2

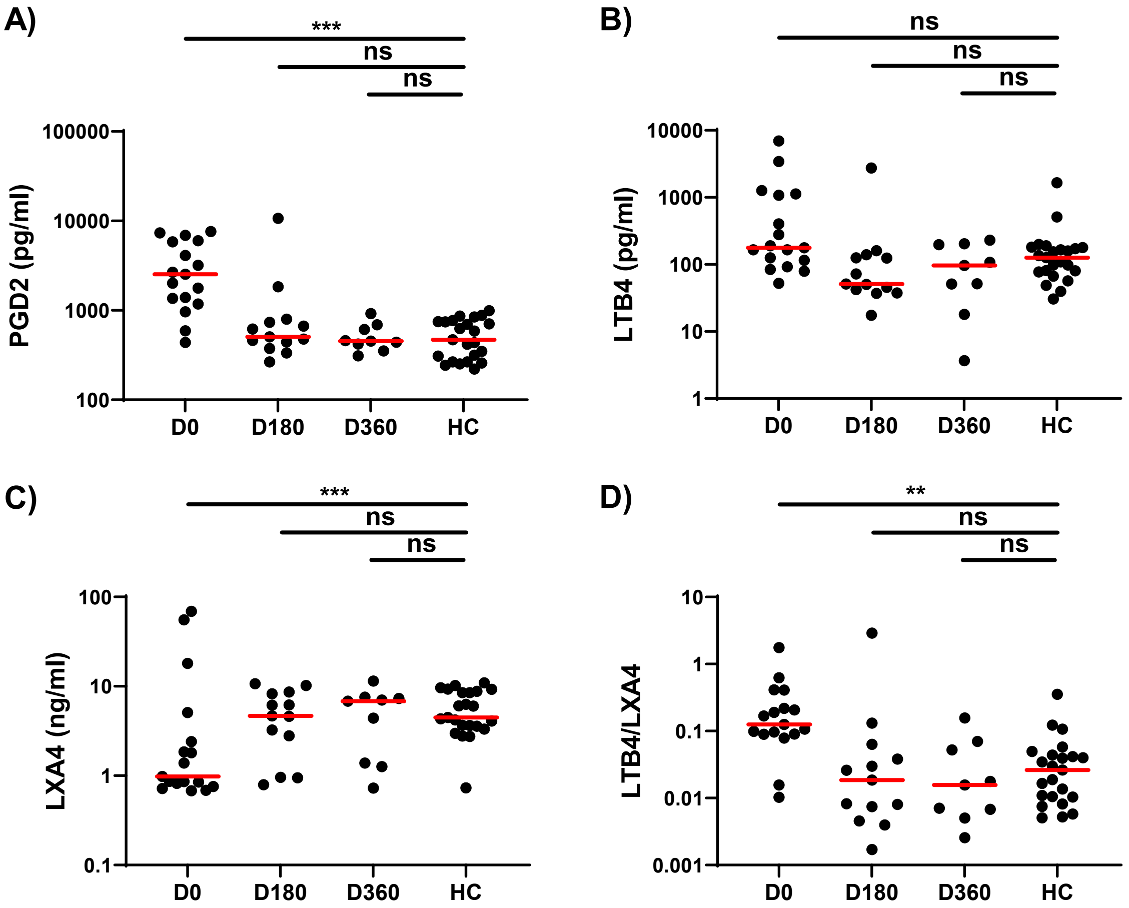

Figure S2. Dysregulated pro- and anti-inflammatory lipid mediators were normalized in 6- and 12-month follow up samples from patients with alcoholic hepatitis (AH) who stopped drinking. Scatter plots showing plasma levels of PGD2 (A), LTB4 (B), and LXA4 (C), and TLB/LXA4 ratio (D) in AH patients at enrollment (D0), 6-month follow-up (D180), 12-month follow-up (D360), and healthy controls (HC). Kruskal-Wallis test with Dunn's correction for comparisons of D0, D180, and D360 with HC. **$p < 0.01$, ***$p < 0.001$. ns, not significant.

Figure S3

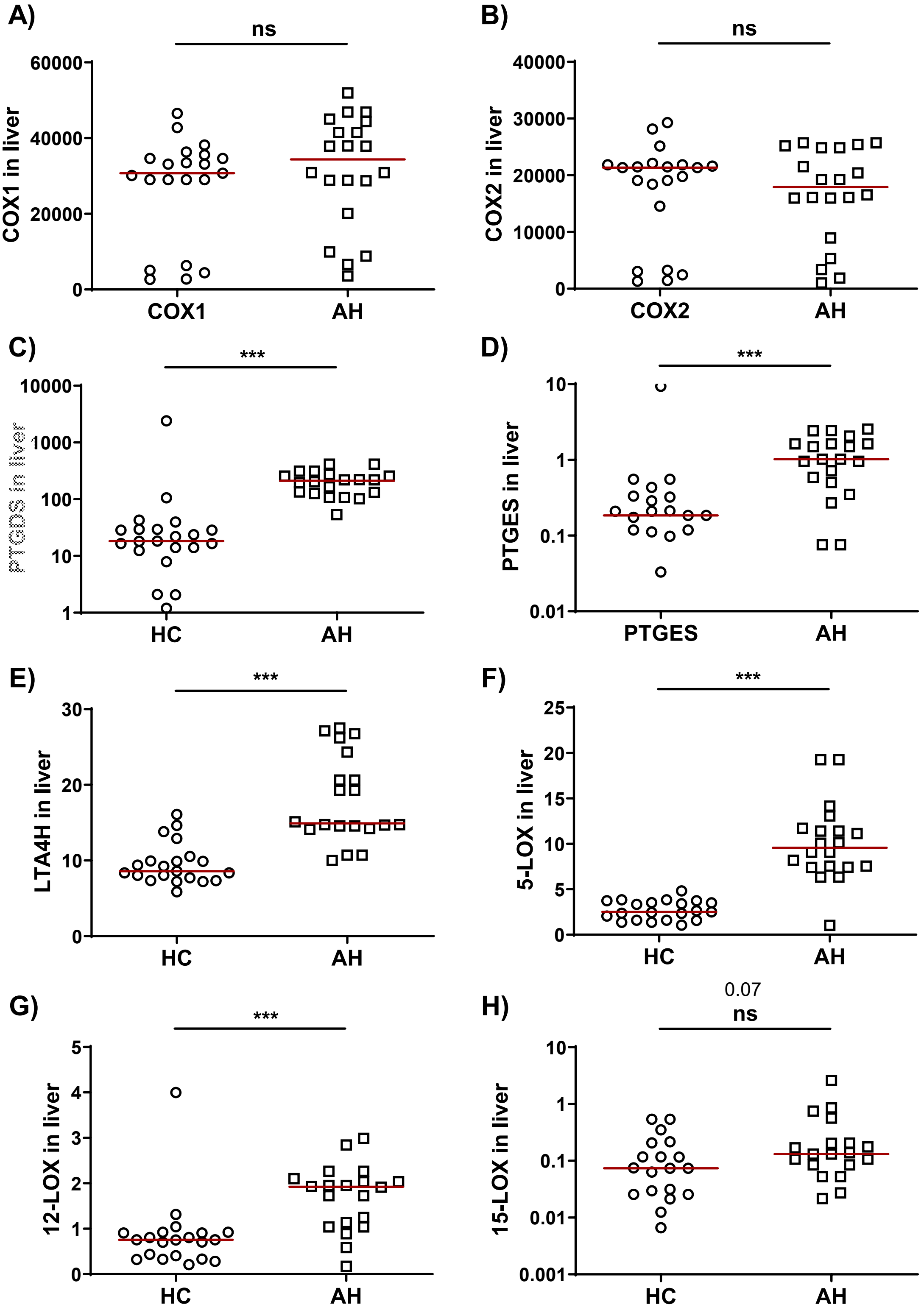

Figure S3. Expression of genes involved in production of lipid mediators in liver tissue from patients with alcoholic hepatitis and healthy controls. Scatter plots showing expression of *COX1* (A), *COX2* (B), *PTGDS* (C), *PTGES* (D), *LTA4H* (E), *5-LOX* (F), *12-LOX* (G), and *15-LOX* (H). RNA expression levels were pooled from liver tissue RNA-seq databases GSE143318, GSE142530, and GSE155907. Data are presented as TPM normalized expression values. TPM, transcripts per kilobase million; HC, healthy controls; AH, patients with alcoholic hepatitis. Two-tailed t test was used to calculate differences between AH and HC. ***$p < 0.001$. ns, not significant.

Figure S4

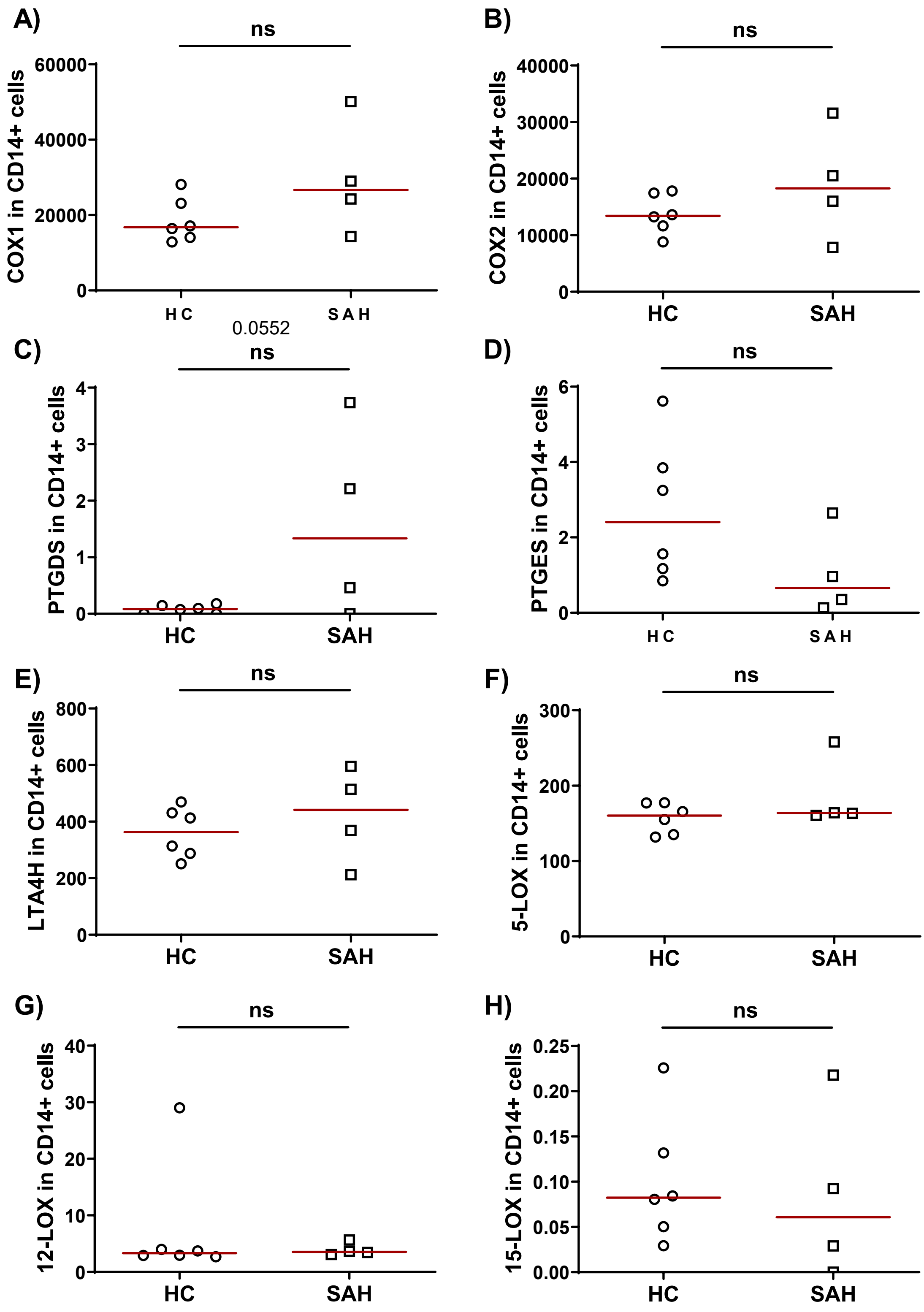

Figure S4. Expression of genes involved in production of lipid mediators in peripheral blood monocytes from patients with severe alcoholic hepatitis (SAH) and healthy controls. Scatter plots showing expression of *COX1* (A), *COX2* (B), *PTGDS* (C), *PTGES* (D), *LTA4H* (E), *5-LOX* (F), *12-LOX* (G), and *15-LOX* (H). RNA expression levels were extracted from RNA-seq database GSE135285. Data are presented as TPM normalized expression values. TPM, transcripts per kilobase million; HC, healthy controls; SAH, patients with severe alcoholic hepatitis. Two-tailed t test was used to calculate differences between AH and HC. ns, not significant.

Figure S5

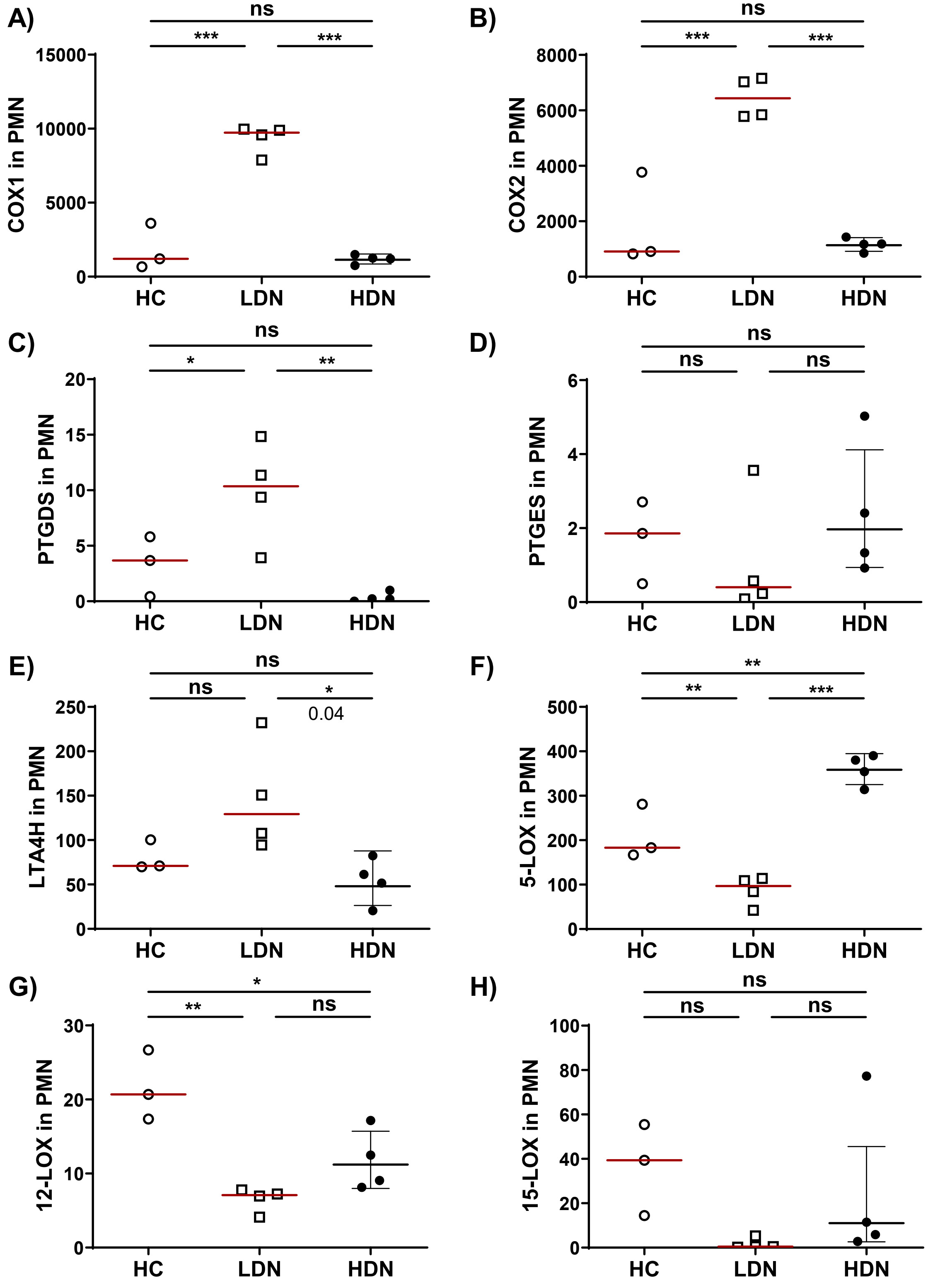

Figure S5. Expression of genes involved in production of lipid mediators in peripheral blood neutrophils (PMN) in patients with alcoholic hepatitis and healthy controls. Scatter plots showing expression of *COX1* (A), *COX2* (B), *PTGDS* (C), *PTGES* (D), *LTA4H* (E), *5-LOX* (F), *12-LOX* (G), and *15-LOX* (H). RNA expression levels were extracted from neutrophil RNA-seq database GSE1710809. Data are presented as TPM normalized expression values. TPM, transcripts per kilobase million; HC, healthy controls; AH, patients with alcoholic hepatitis; LDN, low density neutrophils from AH patients; HDN, high density neutrophils from AH patients; Ordinary one-way ANOVA with Holm-Sidak's multiple comparisons test was used for comparisons among HC, LDN, and HDN. *$p < 0.05$, **$p < 0.01$, ***$p <$ 0.001, ns, not significant.
