# Supplementary material for: Disrupted balance between pro-inflammatory lipid mediators and anti-inflammatory specialized pro-resolving mediators is linked to hyperinflammation in patients with alcoholic hepatitis
Source: Front Immunol. 2024 Nov 21;15:1377236. doi: 10.3389/fimmu.2024.1377236 (PMC11617321; doi:10.3389/fimmu.2024.1377236)
Supplement: Supplementary file 2 [file DataSheet2.pdf]

**Supplemental Table 1. Comparison of the demographic and clinical characteristics of the longitudinal cohort of patients**

| Variables | AH at baseline (n=17) | AH at 6-months (n=13) | AH at 12-months (n=9) | HC (n=23) | *p* value |
|---|---|---|---|---|---|
| **Demographics** | | | | | |
| Age (years) | 45 (33-53) | 40 (32-50) | 45 (37-54) | 42 (30-56) | 0.73 |
| Gender (% Male) | 9 (53) | 8 (62) | 4 (44) | 9 (60) | 0.69 |
| **Clinical parameters** | | | | | |
| Creatinine (mg/dL) | 0.80 (0.69-1.34) | 0.70 (0.57-1.00) | 0.86 (0.60-1.14) | 0.86 (0.73-1.02) | 0.61 |
| Total bilirubin (mg/dL) | 14.3 (7.8-21.9)*** | 1.2 (0.7-4.6)** | 0.7 (0.5-1.4) | 0.4 (0.2-0.5) | **<0.001** |
| ALT (IU/L) | 50 (26-65)*** | 26 (21-41)** | 24 (18-33) | 12 (8-18) | **<0.001** |
| AST (IU/L) | 115 (82-147)*** | 34 (30-72)** | 28 (24-37) | 17 (13-22) | **<0.001** |
| Prothrombin time (INR) | 1.84 (1.62-2.28) | 1.33 (1.09-1.54)## | 1.10 (1.03-1.43)## | | **0.001** |
| MELD score | 25 (20-30) | 10 (8-16)## | 9 (7-13)### | | **<0.001** |
| Neutrophils (X$10^3$/µl) | 8.1 (5.4-17.3) | N/A | N/A | | |
| Platelets (X$10^3$/µl) | 172 (119-229) | 114 (92-156) | 155 (116-219) | | 0.17 |
| BMI | 27.1 (25.4-31.2) | 27.9 (24.6-33.2) | 24.7 (22.6-28.7) | | 0.27 |
| Treated with PDN (# and %) | 9 (53%) | | | | |

**Note:** Data are represented as median and interquartile ranges or number and %. AH, patients with alcoholic hepatitis; HC, healthy controls; ALT, alanine aminotransferase; AST, aspartate aminotransferase; INR, international normalized ratio; MELD, model for end-stage liver disease; BMI, body mass index; N/A, data not available; PDN, prednisone. Kruskal-Wallis test with Dunn's correction was used for comparisons between HC and AH patients at baseline, 6-month, or 12-month follow-up. $^{**}p < 0.01$, $^{***}p < 0.001$. Kruskal-Wallis test with Dunn's correction for comparisons between AH patients at baseline and at 6-month or 12-month follow-up. $^{\#\#}p < 0.01$, $^{\#\#\#}p < 0.001$. Chi-square test for analysis of gender difference. $p < 0.05$ was considered statistically significant (bolded).

**Supplemental Table 2. Numbers of plasma samples with detectable levels of lipid mediators/precursors as measured by LS-MS/MS**

| | | HC (n=10) | AH (n=10) | HDC (n=10) |
|---|---|---|---|---|
| **AA pathway** | Leukotriene B4 | 10 | 10 | 10 |
| | Prostaglandin D2 | 10 | 10 | 10 |
| | Prostaglandin E2 | 10 | 10 | 10 |
| | Lipoxin A4 | 1 | 1 | 0 |
| | Lipoxin B4 | 5 | 1 | 2 |
| **EPA pathway** | 18-HEPE | 10 | 10 | 10 |
| | Resolvin E1 | 1 | 2 | 2 |
| **DHA pathway** | 17-HDHA | 10 | 10 | 10 |
| | Resolvin D1 | 10 | 10 | 10 |
| | Resolvin D2 | 10 | 10 | 10 |
| | Resolvin D3 | 3 | 1 | 0 |
| | Resolvin D4 | 3 | 4 | 3 |
| | Resolvin D5 | 2 | 4 | 1 |
| | Protectin 1 | 1 | 1 | 2 |
| | Protectin DX | 2 | 2 | 1 |
| | 14-HDHA | 10 | 10 | 10 |
| | Maresin 1 | 3 | 1 | 2 |
| | Maresin 2 | 0 | 0 | 0 |

**Note:** HC, healthy controls; AH, patients with alcoholic hepatitis; HDC, heavy drinking controls; AA, arachidonic acid; EPA, eicosatetraenoic acid; HEPE, hydroxyeicosapentaenoic acid; DHA, docosahexaenoic acid; HDHA, hydroxy docosahexaenoic acid.

**Supplemental Table 3. Gender differences in plasma levels of lipid mediators in patients with alcoholic hepatitis**

| | Females (n=13-23) | Males (n=11-22) | *p* value |
|---|---|---|---|
| **PLMs** | | | |
| Leukotriene B4 (pg/ml) | 207.1 (98.1-1189) | 271.2 (140.8-801) | 0.74 |
| Prostaglandin D2 (pg/ml) | 1519 (921.3-2405.5) | 1771.9 (775.5-2852.5) | 0.82 |
| Prostaglandin E2 (pg/ml) | 115.1 (59.9-155.6) | 139.1 (41.7-239.6) | 0.42 |
| **SPMs** | | | |
| Lipoxin A4 (ng/ml) | 1.2 (0.7-1.9) | 1.1 (0.7-1.5) | 0.58 |
| Resolvin D2 (pg/ml) | 126.5 (72.7-180.3) | 89 (60.7-129.8) | 0.26 |
| Resolvin E1 (pg/ml) | 818.6 (321.7-1645.6) | 574.1 (366.3-1056.4) | 0.54 |
| Maresin 1 (pg/ml) | 190.3 (111-225.9) | 101.1 (57.8-207.1) | 0.08 |

**Note:** Data are represented as median and interquartile ranges. PLMs, proinflammatory lipid mediators; SPMs, specialized pro-resolving mediators. Mann Whitney test was used to compare levels in male versus female patients with alcoholic hepatitis.

**Supplemental Table 4. Comparison of plasma levels of lipid mediators in patients with alcoholic hepatitis treated versus untreated with prednisone**

| | Untreated (n=7-17) | Treated (n=16-34) | *p* value |
|---|---|---|---|
| **PLMs** | | | |
| Leukotriene B4 (pg/ml) | 190.8 (127.7-739.5) | 270.3 (98.1-1147.6) | 0.91 |
| Prostaglandin D2 (pg/ml) | 1659.9 (862.6-2901.7) | 1550.7 (894.8-2458.5) | 0.73 |
| Prostaglandin E2 (pg/ml) | 191.8 (90.5-343.1) | 116.8 (43.1-138.7) | 0.10 |
| **SPMs** | | | |
| Lipoxin A4 (ng/ml) | 1.1 (0.7-2) | 1.2 (0.6-1.6) | 0.66 |
| Resolvin D2 (pg/ml) | 107.5 (66.3-156.7) | 124.5 (73.2-176.4) | 0.61 |
| Resolvin E1 (pg/ml) | 858.4 (434.6-1700.4) | 574.1 (275.6-1198.2) | 0.14 |
| Maresin 1 (pg/ml) | 102.2 (55.2-162.0) | 195.7 (74.8-226.2) | 0.17 |

**Note:** Data are represented as median and interquartile ranges. PLMs, proinflammatory lipid mediators; SPMs, specialized pro-resolving mediators. Mann Whitney test was used to compare levels in patients with alcoholic hepatitis treated versus untreated with prednisone.

**Supplemental Table 5. Comparison of plasma levels of lipid mediators in deceased and surviving patients with alcoholic hepatitis during the 12-month follow-up**

| | Deceased (n=4-7) | Survivors (n=20-49) | *p* value |
|---|---|---|---|
| **PLMs** | | | |
| Leukotriene B4 (pg/ml) | 214.6 (98.1-1561.9) | 238.7 (120.5-1058.5) | 0.82 |
| Prostaglandin D2 (pg/ml) | 1321.3 (628.4-1857) | 1550.7 (864.4-2663.5) | 0.39 |
| Prostaglandin E2 (pg/ml) | 100.1 (46.4-236.8) | 120.4 (47.8-189.1) | 0.92 |
| **SPMs** | | | |
| Lipoxin A4 (ng/ml) | 0.8 (0.6-1.3) | 1.2 (0.7-1.8) | 0.33 |
| Resolvin D2 (pg/ml) | 102.4 (77.6-155.9) | 113 (63.9-171.6) | >0.99 |
| Resolvin E1 (pg/ml) | 323.6 (280.8-652.9) | 858.4 (388.4-1449.5) | 0.12 |
| Maresin 1 (pg/ml) | 222.3 (148.7-228.2) | 116.2 (59.5-219.7) | 0.16 |

**Note:** Data are represented as median and interquartile ranges. PLMs, proinflammatory lipid mediators; SPMs, specialized pro-resolving mediators. Mann Whitney test was used to compare levels in deceased vs surviving patients with alcoholic hepatitis.

**Supplemental Table 6. Comparison of plasma levels of proinflammatory mediators related with lipid mediators in patients with alcoholic hepatitis in 3 study groups at baseline**

| | | HC (n=16-19) | AH (n=28-53) | HDC (n=19-28) | *p* value |
|---|---|---|---|---|---|
| **Inflammation markers** | CRP (μg/ml) | 1.4 (0.4-9.6)### | 41.2 (27.5-75.5)*** | 8.8 (3.4-27.4) | **<0.001** |
| | LPS (pg/ml) | 0.01 (0.01-0.01)### | 0.043 (0.01-0.40)*** | 0.01 (0.01-0.01 | **<0.001** |
| | LBP (μg/ml) | 6.7 (4-8.1)### | 13.5 (8.3-18.8)** | 8.5 (6.2-11.3) | **<0.001** |
| | sCD14 (ng/ml) | 1185.7 (1078.2-1689.3)### | 2270.5 (1705.3-3626.7)* | 1798 (1236-2125) | **<0.001** |
| | sCD163 (ng/ml) | 531.2 (417.4-732.4)### | 4646.5 (3226.4-5779.3)*** | 503 (301-561) | **<0.001** |
| **Cytokines** | IFN-α (pg/ml) | 0.7 (0.2-1.2) | 0.9 (0.3-2)* | 0.2 (0.2-0.6) | **0.045** |
| | IL-1RA (pg/ml) | 17.8 (17.8-17.8)### | 518.1 (17.8-1599.3)** | 17.8 (17.8-353.1) | **<0.001** |
| | IL-8 (pg/ml) | 1.2 (1.2-4.1)### | 197.5 (134.3-366.2)*** | 1.2 (1.2-1.2) | **<0.001** |
| | IL-10 (pg/ml) | 0.7 (0.4-0.9)### | 3.1 (1.4-4.3)*** | 1.0 (0.8-1.1) | **<0.001** |
| | IL-13 (pg/ml) | 0.1 (0.1-0.1)## | 0.1 (0.1-1.1)* | 0.1 (0.1-0.1) | **0.003** |
| | TNF−α (pg/ml) | 6.1 (5.1-8)### | 9.9 (8.3-17.8)** | 7.7 (6.8-8.6) | **<0.001** |
| **Chemokines** | IP10 (pg/ml) | 8 (5.1-14.3)### | 22.4 (14.1-43.2)*** | 6.2 (3.7-8.4) | **<0.001** |
| | MIP-1α (pg/ml) | 16.5 (9.6-36.3) | 26.9 (15-57.2)*** | 8.8 (7.7-13.8) | **<0.001** |
| | MIP-1β (pg/ml) | 71.8 (44.7-109.4) | 96.3 (71.1-135.7)* | 61.5 (52.2-95.6) | **0.010** |
| | RANTES (pg/ml) | 60.5 (23.1-82.9) | 86.3 (48.9-129.3)*** | 35.4 (25.9-51.2) | **<0.001** |
| **Growth factors** | FGF-2 (pg/ml) | 2.5 (2.5-2.5) | 2.5 (2.5-7.6) | 2.5 (2.5-2.5) | 0.375 |
| | HGF (pg/ml) | 42.2 (19.3-51.1)### | 891.1 (440.6-1327.5)*** | 20.7 (13-53.3) | **<0.001** |
| | VEGF (pg/ml) | 94.3 (41.6-154.7)## | 168.7 (124.1-529.4)** | 98.5 (69.6-139.5) | **<0.001** |

**Note:** Data are represented as median and interquartile ranges. HC, healthy controls; AH, patients with alcoholic hepatitis; HDC, heavy drinking controls. Kruskal-Wallis test with Dunn's correction for pairwise comparisons of continuous variables among HCs, AH patients, and HDCs. ##$p < 0.01$, ###$p < 0.001$ for comparison between AH patients and HC; *$p < 0.05$, **$p < 0.01$, ***$p < 0.001$ for comparison between AH patients and HDC. $p < 0.05$ was considered statistically significant **(bolded)**.
